# Parietal memory network and default mode network in first‐episode drug‐naïve schizophrenia: Associations with auditory hallucination

**DOI:** 10.1002/hbm.24923

**Published:** 2020-02-29

**Authors:** Qian Guo, Yang Hu, Botao Zeng, Yingying Tang, Guanjun Li, Tianhong Zhang, Jinhong Wang, Georg Northoff, Chunbo Li, Donald Goff, Jijun Wang, Zhi Yang

**Affiliations:** ^1^ Shanghai Key Laboratory of Psychotic Disorders, Shanghai Mental Health Center Shanghai Jiao Tong University School of Medicine Shanghai China; ^2^ Brain Science and Technology Research Center Shanghai Jiao Tong University Shanghai China; ^3^ Department of Early Psychotic Disorders, Shanghai Mental Health Center Shanghai Jiao Tong University School of Medicine Shanghai China; ^4^ Department of Psychiatry Qingdao Mental Health Center Qingdao China; ^5^ Department of Medical Imaging, Shanghai Mental Health Center Shanghai Jiao Tong University School of Medicine Shanghai China; ^6^ University of Ottawa Brain and Mind Research Institute, and Mind Brain Imaging and Neuroethics Royal's Institute of Mental Health Research University of Ottawa Ottawa Ontario Canada; ^7^ Key Laboratory for the Genetics of Developmental and Neuropsychiatric Disorders, Bio‐X Institutes, Ministry of Education Shanghai Jiao Tong University Shanghai China; ^8^ Institute of Psychology and Behaviour Science Shanghai Jiao Tong University Shanghai China; ^9^ Department of Psychiatry New York University School of Medicine New York New York

**Keywords:** auditory hallucination, default mode network, functional connectivity, parietal memory network

## Abstract

Atypical spontaneous activities in resting‐state networks may play a role in auditory hallucinations (AHs), but networks relevant to AHs are not apparent. Given the debating role of the default mode network (DMN) in AHs, a parietal memory network (PMN) may better echo cognitive theories of AHs in schizophrenia, because PMN is spatially adjacent to the DMN and more relevant to memory processing or information integration. To examine whether PMN is more relevant to AHs than DMN, we characterized these intrinsic networks in AHs with 59 first‐episode, drug‐naïve schizophrenics (26 AH+ and 33 AH−) and 60 healthy participants in resting‐state fMRI. We separated the PMN, DMN, and auditory network (AN) using independent component analysis, and compared their functional connectivity across the three groups. We found that only AH+ patients displayed dysconnectivity in PMN, both AH+ and AH− patients exhibited dysfunctions of AN, but neither patient group showed abnormal connectivity within DMN. The connectivity of PMN significantly correlated with memory performance of the patients. Further region‐of‐interest analyses confirmed that the connectivity between the core regions of PMN, the left posterior cingulate gyrus and the left precuneus, was significantly lower only in the AH+ group. In exploratory correlation analysis, this functional connectivity metric significantly correlated with the severity of AH symptoms. The results implicate that compared to the DMN, the PMN is more relevant to the AH symptoms in schizophrenia, and further provides a more precise potential brain modulation target for the intervention of AH symptoms.

## 1 INTRODUCTION

Approximately 60–90% of schizophrenia patients experience auditory hallucinations (AHs) through their life course of illness (Saha, Chant, Welham, & McGrath, 2005). AHs are referred to the perception of sound in the absence of an external stimulus that substantially impair quality of life. As AHs usually occur intrusively to ongoing thoughts, it has been assumed that atypical resting‐state brain activity may give rise to hallucinatory experiences (Northoff & Qin, 2011).

Aberrant resting‐state activity in the default mode network (DMN) has been consistently reported in psychotic disorders (Alderson‐Day, McCarthy‐Jones, & Fernyhough, 2015), especially for schizophrenia (Rotarska‐Jagiela et al., 2010). A prevailing hypothesis has indicated that abnormal modulation of auditory cortex by part of DMN in resting‐state may cause one to take internal brain activities as external stimuli (Northoff, 2014). However, findings regarding whether the abnormality of DMN involves in AHs are mixed. Jardri, Thomas, Delmaire, Delion, and Pins (2013) investigated the “real rest” periods in 20 adolescents with a brief psychotic disorder, and associated hallucinations with disengagement and spatial instability of DMN. In contrast, Wolf et al. (2011) failed to find abnormality of DMN, but reported functional connectivity alterations in the precuneus and posterior cingulate in AHs. Further, van Lutterveld, Diederen, Otte, and Sommer (2014) found increased connectivity in the temporal cortices and the posterior cingulate/precuneus in a sample of nonclinical participants with AHs. As the function and boundary of DMN is still in debate (Buckner & DiNicola, 2019), the above heterogeneous results call for investigations into the connectivity pattern of the precuneus/PCC regions to reveal their contributions to AHs.

With respect to cognitive roles, the DMN is commonly associated with self‐referential processing and recollection of autobiographical memories (Buckner, Andrews‐Hanna, & Schacter, 2008). These functions cannot fully explain how the internal mentation is perceived as externally generated in the symptom of AH. On the other hand, accumulated evidences have indicated that failure of memory‐based reality monitoring, which determine the memory source from external perceived or internal imagined events (Garrison, Bond, Gibbard, Johnson, & Simons, 2016), may result in symptom of AH in schizophrenia. Brain areas such as the precuneus/PCC are highly related to source memory or judgment (Garrison et al., 2016; Guidotti, Tosoni, Perrucci, & Sestieri, 2019). A transcranial magnetic stimulation study has provided further support for this link by showing that inhibiting activity in the precuneus decreased the performance in source memory task (Bonnì et al., 2015).

Echoing the memory monitoring theory, a resting‐state network consisting of the precuneus, the middle/posterior cingulate cortex, and the bilateral inferior parietal lobules, which is referred as parietal memory network (PMN, Gilmore, Nelson, & McDermott, 2015), has been demonstrated to involve in “familiarity labeling” in memory and learning (Nelson, Arnold, Gilmore, & McDermott, 2013). The above cognitive‐pathological theory of AHs make a potentially more direct link between the memory dysfunction in AHs and the “familiarity labeling” function of the PMN. Therefore, we hypothesize that deficits in PMN may contribute to AHs.

The PMN is adjacent to the DMN, and there have been evidence from multiple aspects to support the functional segregation of the two networks. Data from task‐fMRI studies demonstrated that PMN could be associated with familiarity processing (Gilmore et al., 2015), and is disassociated from the DMN in different task conditions (Chen, Gilmore, Nelson, & McDermott, 2017). This argument does not conflict with the classical view of DMN, because many of the widely used resting‐state functional network parcellations do not attribute the core regions of the PMN to DMN (Doucet et al., 2011; Power et al., 2011; Yeo et al., 2011). Our previous works have demonstrated that the two networks exhibit different trends in individual variability across the lifespan (Yang et al., 2014) and that the functional segregation of the two networks is robust across different algorithms (Hu et al., 2016). Using a movie‐watching task, we further showed that the activity levels of DMN and PMN were inversely modulated by watching normal and scrambled versions of a movie (Deng et al., 2019). Given these findings, in this study, we considered the PMN and DMN as separated functional networks and examined their abnormality relevant to AH.

Currently, no study has investigated the role of PMN in the pathophysiology of AHs. In this study, using a relatively large sample of first‐episode, drug‐naïve schizophrenia patients and matching healthy controls, we aimed to separate the PMN from DMN in resting‐state and examine their relevance to AHs. Functional connectivity revealed by both independent component analysis (ICA) and region‐of‐interest (ROI) analyses were compared across the patients with AHs, patients without AHs, and healthy controls. While the interaction between auditory network (AN) and DMN was proposed as a possible pathological cause of AH, no research has investigated the relationship between PMN and AN in schizophrenia with AHs up to date. The functional connectivity of AN as well as its interaction with PMN and DMN were also examined to echo the previous findings (Northoff, 2014).

## 2 METHODS

### 2.1 Participants

Sixty‐five healthy controls (HCs), 36 schizophrenic or schizophreniform patients without AHs (AH−), and 29 schizophrenic or schizophreniform patients with AHs (AH+) participated in this study. All participants were recruited from Shanghai Mental Health Center, Shanghai, China. AH+ patients were recruited into the study if they reported AHs at least one episode per day based on the frequency item of the AH rating scale (AHRS; Hoffman et al., 2003). AH− patients were included if they reported no experience of AHs during their courses of illness. The healthy controls were recruited from local communities in Shanghai. Written informed consent was obtained from each participant or the participant's guardian before data acquisition. This study was approved by the Local Research Ethics Committee.

The inclusion criteria for the AH+ and AH− groups were (a) consensus diagnoses of first‐episode schizophrenia or schizophreniform disorder assigned by two psychiatrists, according to the Diagnostic and Statistical Manual of Mental Disorders, Fourth Edition (DSM‐IV), on the basis of Structured Clinical Interview for DSM‐IV; (b) medication‐naïve; (c) education level higher than primary school and capability of finishing the tests; (d) age from 15 to 40. The exclusion criteria were (a) clinically unstable to finish the assessments; (b) presence of another axis I psychiatric disorder; (c) rated seven or higher in the Calgary Depression Scale for Schizophrenia (CDSS); (d) history of suicidal behavior; (e) history of antipsychotic medication; (f) history of substance abuse; (g) pregnancy; (h) history of serious physical diseases; (i) unsuitability for MRI scans, for instance, having metal implants. The HC group was matched with the AH− and AH+ groups for age, gender, and education level. None of the HCs had a positive family history for any psychiatric disorder. Healthy participants were screened with the Chinese version of the MINI, Version 5.0 (Sheehan et al., 1998; Si et al., 2009) and excluded if they met criteria for any mental disorder according to the DSM‐IV or had a history of suicidal behaviors, severe physical diseases, pregnancy, taking any antipsychotic drugs, or substance abuse.

### 2.2 Clinical and cognitive measurements

Clinical symptoms were assessed by a trained psychiatrist， using the 24‐item Brief Psychiatric Rating Scale (BPRS) Expanded Version (Ventura et al., 1986) and the Scale for Assessment of Negative Symptoms (SANS) (Andreasen, 1989). According to Ruggeri et al. (2005), we used subscales of grandiosity, suspiciousness, hallucinations, unusual thought content and conceptual disorganization to define positive symptom of BPRS. All assessments were conducted before drug medication for patients. The severity of AHs was evaluated using a Chinese version of AHRS (Hoffman et al., 2003), which measures frequency, reality, loudness, number of voices, length, attention salience, and level of distress caused by the AHs. The AHRS was firstly translated into Chinese and then translated back to English by professionals, ensuring its accuracy. Additionally, the Chinese version of AHRS has been published in the textbook of *Physical Therapy of Mental Disorders* in Chinese by our research team (Wang, 2012). Duration of untreated psychosis (DUP) was acquired for each patient. DUP was defined as the length of time an individual was affected by the psychotic symptoms without receiving medication treatment and was acquired at the first examination. The Chinese version of MATRICS Consensus Cognitive Battery (MCCB) was used for the assessment of cognitive functions (Nuechterlein et al., 2008). MCCB includes seven neurocognitive domains: speed of processing, attention and vigilance, working memory, verbal learning, visual learning, reasoning and problem solving, and social cognition. A composite score was then calculated based on scores of the above domains.

### 2.3 MRI data acquisition

All participants completed functional and structural MRI on a 3.0 T Siemens Verio MRI scanner (Siemens Medical Solutions, Erlangen, Germany) at Shanghai Mental Health Center. To rule out the effect of the medication, the patients did not take medicine before the MRI scanning, and they received regular pharmacological treatments after the scan. The time from enrollment was usually 2–3 days and no more than a week. After three‐plane localizer, an anatomical scan was acquired with a T1‐weighted magnetization prepared rapid gradient echo sequence (192 sagittal slices, echo time TR/TE/TI = 2,300/2.96/900 ms, flip angle = 9°, FOV = 256 mm, matrix = 256 × 240, slice thickness/gap = 1.0/0.0 mm). An 8′30″ resting‐state fMRI (rs‐fMRI) was acquired subsequent to T1 image with an echo‐planar imaging sequence (45 axial slices, acquired from inferior to superior in an interleaved manner, TR/TE = 3,000/30 ms, flip angle = 85°, FOV = 216 mm, matrix = 72 × 72, slice thickness/gap = 3.0/0.0 mm, 170 volumes). Subjects were instructed to close their eyes and remain awake during the MRI scan. Awakeness during MRI acquisition was confirmed in a brief interview after the scanning.

### 2.4 MRI preprocessing and quality control

After quality control of raw data, T1‐weighted structural images were bias corrected and segmented into gray matter, white matter, and cerebrospinal fluid using Volbrain (Version 1.0; Manjon & Coupe, 2016) and then nonlinearly transformed into MNI152 standard space using ANTs (Version 2.2; Tustison et al., 2014). As for rs‐fMRI images, the following steps were applied using FSL (Version 5.0.11; Jenkinson, Beckmann, Behrens, Woolrich, & Smith, 2012) and AFNI (Cox, 2012; Version 17.2.07): (a) discarding the first 10 volumes; (b) head motion correction; (c) slice time correction; (d) registration to corresponding structural images with boundary‐based registration (Greve & Fischl, 2009); (e) scaling the data to a global mean intensity of 10,000; (f) band‐pass temporal filtering (0.01–0.1 Hz). The preprocessed fMRI images were finally transformed into MNI152 space and resampled into 3 × 3 × 3 mm^3^. Of note, for ICA‐based functional connectivity analysis, the common nuisance regression was omitted by considering the facts that previous studies have demonstrated that nuisance regression could potentially remove brain activity signals besides noises (Bright & Murphy, 2015) and ICA could split noises from brain activities in a data‐driven manner and thus mitigate the influence of noises (Du et al., 2016). For ROI‐based functional connectivity analysis, nuisance regression was additionally applied before temporal filtering. The nuisance regressors included mean signals of white matter and ventricles, Friston's 24‐parameter motion model and motion outliers. The white matter and ventricle masks were created by combining individual‐level segmentation results and tissue priors from Harvard‐Oxford Subcortical Atlas in FSL (https://fsl.fmrib.ox.ac.uk/fsl/fslwiki/Atlases). The volumes with frame‐wise displacement (FD) higher than 0.5 mm were treated as motion outliers.

Quality of brain extraction, tissue segmentation, and spatial registration was visually inspected. Images of five HC, three AH−, and two AH+ were excluded for further analysis due to poor brain extraction or spatial registration. The head motions in the rs‐fMRI data were measured using the maximum translation/rotation, mean frame‐wise displacement (meanFD; Power, Barnes, Snyder, Schlaggar, & Petersen, 2012), and the ratio of motion outliers (motion corrupted volumes divided by the total volumes). The maximum translation/rotation <3 mm/3°, meanFD <0.5 mm, and ratio of motion outliers <0.2 were ensured in all subjects. Furthermore, one AH+ subject was excluded whose total hallucination symptom scored only three and was lower than two *SD*s from the group mean. Therefore, 60 HC, 33 AH−, and 26 AH+ entered the final analysis.

### 2.5 Independent component analysis

The preprocessed rs‐fMRI images of all subjects were temporally concatenated and decomposed into a set of group‐level independent components (ICs) using the MELODIC module of the FSL package (Beckmann, DeLuca, Devlin, & Smith, 2005). The number of components was automatically estimated to be 53. ICs representing AN, DMN, and PMN were selected according to the spatial concordance of the core regions to the existing network atlas (Hu et al., 2016; Yeo et al., 2011). Dual regression was applied to obtain individual IC maps and time courses for every subject (Beckmann, Mackay, Filippini, & Smith, 2009). In brief, for each subject, the spatial maps of the group‐level ICs were used as regressors, and their contributions to the subject's rs‐fMRI data were estimated using a linear model, yielding time courses of the ICs. These time courses were then used as regressors, and their contributions to the same subject's rs‐fMRI dataset were estimated, yielding a set of spatial maps. Each map represented a subject‐specific resting‐state network that is aligned to the corresponding group‐level IC. The individual IC maps were further smoothed with a 6 mm FWHM isotropic Gaussian kernel before statistical analysis.

### 2.6 Statistical analyses

Statistical analyses of demographic, clinical, and cognitive data were examined using R (Version 3.5.2; R Core Team, 2018). ANOVA (analysis of variance) models were used to compare normally distributed variables across groups, and Chi‐square tests were used for categorical variables.

For rs‐fMRI data, we compared ICs of interest across the three groups in two ways: (a) IC maps for the AN, DMN, and PMN were thresholded at a local false discovery rate of *p* < .05 using Gaussian mixture modeling (Beckmann & Smith, 2004) to reflect the core regions of the AN, DMN, and PMN. The mean of the voxel‐wise weights within the core regions were compared across the three groups (Mingoia et al., 2012). This metric reflects the overall functional connectivity of a network because the weight of a voxel indicates its functional connectivity with the core regions in the given IC. To rule out the averaging effect on network analysis, we delineated the clusters with a minimum of 200 voxels and treated these clusters as the major nodes for each network. The mean of the voxel‐wise weights within each node were compared across the three groups. (b) To reveal foci with significant group differences, a nonparametric permutation *F*‐test (5,000 permutations) was applied to compare voxel‐wise weights of AN, DMN, and PMN across the three groups (Winkler, Ridgway, Webster, Smith, & Nichols, 2014). Of note, voxel‐wise comparisons were restricted to networks with significant group difference identified in the network‐level analysis.

The multiple comparison correction to control family‐wise error rate (FWE) was conducted with a threshold‐free cluster enhancement approach (TFCE; Smith & Nichols, 2009). The TFCE method integrates the peak and extent information to increase the statistical sensitivity and avoids the necessity to set a cluster‐forming threshold as the classical cluster‐wise multiple comparison correction techniques. Post‐hoc analysis was performed on the mean of the significant clusters revealed in the voxel‐wise ANOVA. The mean weights of the clusters (reflecting the overall functional connectivity of each cluster) were compared across groups and correlated with the clinical and cognitive measures such as MCCB and AHRS in the patient groups.

We used region‐of‐interest approach to validate the above findings and further explore the between‐network interactions. The significant clusters obtained above were used as ROIs, and their mean time‐series were extracted. Functional connectivity among the ROIs was measured by Pearson's correlation coefficients (Fisher‐*Z* transformed). These functional connectivity metrics were compared between groups and correlated with clinical and cognitive measures.

## 3 RESULTS

### 3.1 Demographic, clinical, and cognitive characteristics

Demographic data did not differ in age, gender, or education years among AH−, AH+ patients and healthy participants. For clinical assessments, AH− and AH+ groups showed no significant difference in BPRS total score, score of positive symptoms of BPRS, and score of SANS. The HC group displayed significantly higher performance than both AH− and AH+ groups in all domains of MCCB except social cognition, which exhibited no group difference (see Table S1). The two patient groups displayed no significant difference in cognitive assessments. The demographic and clinical characteristics of participants enrolled in the final analysis are presented in Table 1.

**Table 1:** Demographic and clinical characteristics for schizophrenic subgroups and healthy controls

Characteristics	Schizophrenic subgroups		Healthy controls (*N* = 60)^a^	Group comparison *F*/*t* (*p*) value
	AH− patients (*N* = 33)^a^	AH+ patients (*N* = 26)^a^		
Gender (male/female)	12/21	12/14	29/31	*χ* ^2^ (2) = 1.27 (.53)
Age (years)	24.67 (7.39)	27.08 (6.30)	25.03 (6.41)	*F*(2,116) = 1.11 (.33)
Education years	12.82 (2.83)	12.42 (2.97)	12.70 (2.82)	*F*(2,116) = 0.15 (.87)
Duration of untreated psychosis (weeks)	36.42 (41.96)	32.19 (33.18)	−	*t*(57) = 0.42 (.68)
Auditory hallucination rating scale	−	26.42 (6.70)	−	NA
Brief psychiatric rating scale				
Total score	45.79 (8.38)	46.88 (8.33)	−	*t*(57) = −0.50 (.62)
Positive symptom score	14.94 (4.30)	16.12 (4.20)	−	*t*(57) = −1.05 (.30)
Scale for assessment of negative symptoms	21.97 (12.79)	17.54 (11.27)	−	*t*(57) = 1.39 (.17)

Abbreviations: AH−， schizophrenic or schizophreniform patients without auditory hallucinations; AH+, schizophrenic or schizophreniform patients with auditory hallucinations.

a Data are presented as mean (*SD*).

### 3.2 Network‐wise functional connectivity analysis

**Figure 1:**
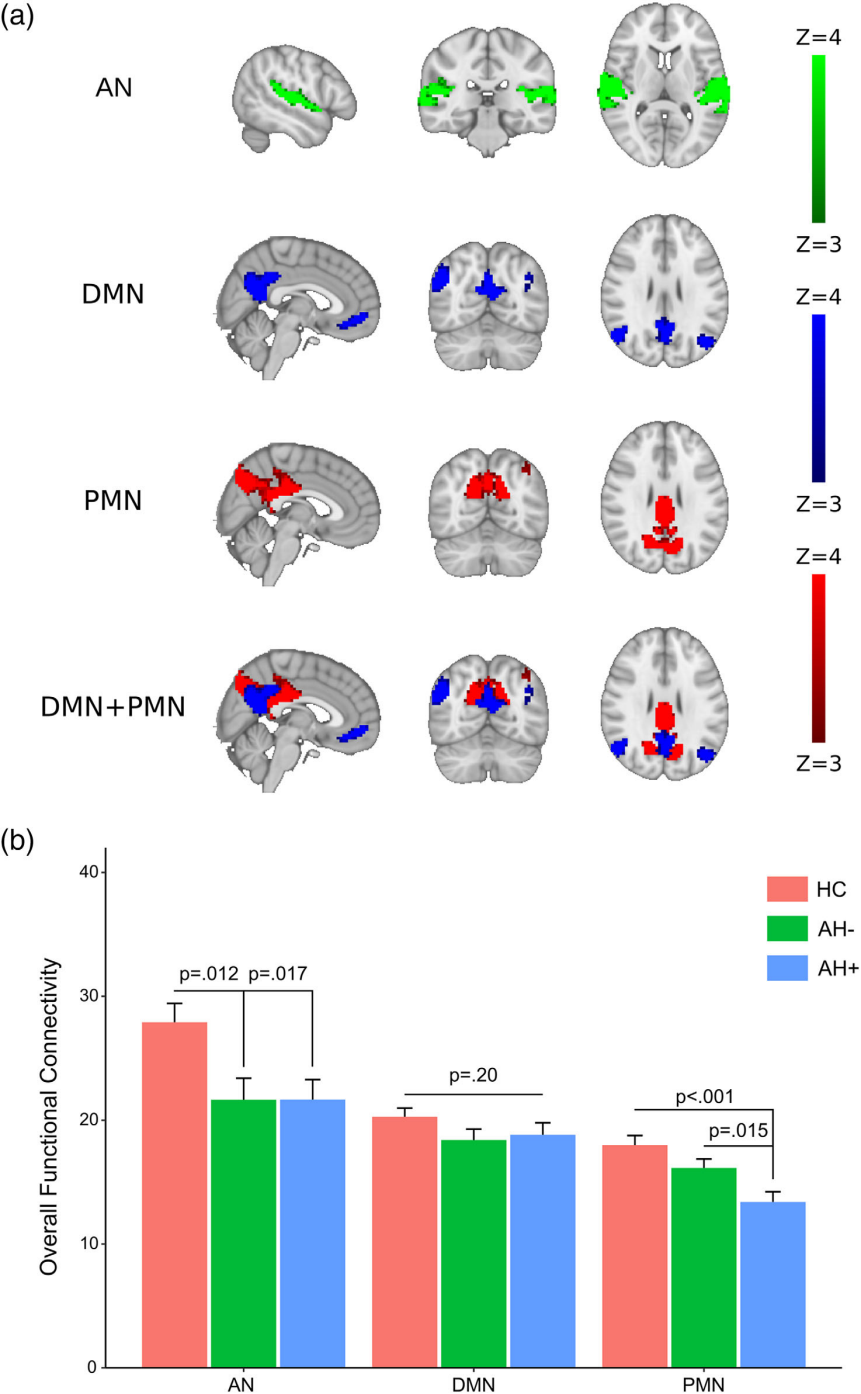
(a) The group‐level component maps representing auditory network (AN), default mode network (DMN), and parietal memory network (PMN). The component maps are converted into *Z*‐scores and thresholded at *Z* = 3 to reveal the core regions. In the last row, the DMN is placed above the PMN to show the adjacent but distinct patterns between the DMN and PMN. The brains are displayed in radiological orientation (i.e., left is right). (b) The group difference in the overall functional connectivity of AN, DMN, and PMN. Only PMN shows significant difference between AH+ and both the HC and AH− groups, while the DMN does not show significant difference across groups. Both AH− and AH+ show significant difference from HC in the overall functional connectivity of AN. AH, auditory hallucination; HC, healthy control

The group‐level component maps representing AN, DMN, and PMN are presented in Figure 1a. As expected and in general, the AN was composed of bilateral superior temporal gyrus, the DMN was composed of posterior cingulate/precuneus, medial prefrontal cortex, and bilateral inferior parietal lobules, and the PMN was composed of precuneus, middle/posterior cingulate, and inferior parietal lobules.

To validate our result that the PMN and DMN were separated networks in the ICA analysis, we utilized the data of HC group to calculate the voxel‐wise functional connectivity in DMN and PMN, and examined the averaged connectivity pattern of the two networks. As demonstrated in Figure S1, the core regions of PMN and DMN were spatially adjacent, but the average connectivity strength within PMN and within DMN was significantly higher than that between the two networks (*p* < .0001).

As presented in Figure 1b, the three groups showed significant differences in the overall functional connectivity of PMN (*F*[2,116] = 7.11, *p* = .0012) and AN (*F*[2,116] = 5.12, *p* = .0074), but not DMN (*F*[2,116] = 1.63, *p* = .20). Post‐hoc analysis revealed that the overall functional connectivity of PMN was significant lower in the AH+ group, when compared to HC group (*t*[84] = 3.51, *p* < .001) and AH− group (*t*[57] = 2.52, *p* = .015), suggesting impaired functional connectivity of PMN in AH+. The difference between AH− and HC was not significant (*t*[91] = 1.55, *p* = .12). As for the overall functional connectivity of AN, AH− did not significantly differ from AH+ group (*t*[57] = 0.0068, *p* = .99), and both groups exhibited significantly lower functional connectivity than HC (for AH−, *t*(91) = 2.57, *p* = .012; for AH+, *t*(84) = 2.44, *p* = .017).

Further comparisons on the major nodes for each network also obtained similar results to the above findings. As shown in Figure S2 and Table S2, the pivotal nodes of DMN, no matter anterior or posterior parts, did not yield any statistical group difference, while the middle/posterior cingulate and precuneus nodes of PMN as well as bilateral AN nodes still exhibited significant difference.

### 3.3 Voxel‐wise functional connectivity analysis

**Figure 2:**
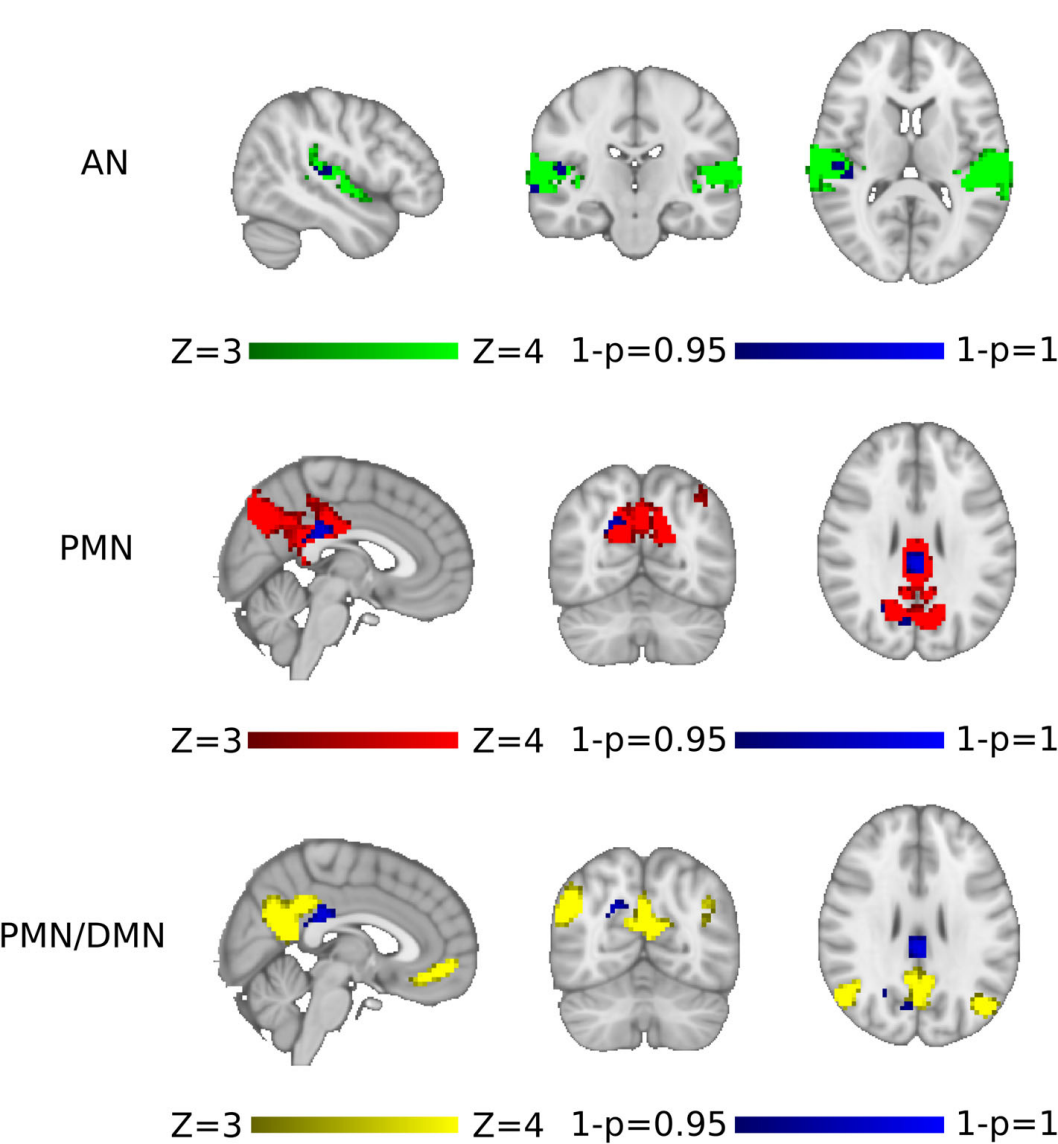
The voxel‐wise difference revealed in the *F*‐test across the three groups for AN and PMN. The significant difference (in blue) is placed above group‐level AN (in green) and PMN maps (in red). In the third row, the significant difference of PMN is placed above the DMN (in yellow) to show that the difference is not located inside the core regions of DMN. These significant clusters are defined as ROIs for further analysis. The brains are displayed in radiological orientation (i.e., left is right). AN, auditory network; DMN, default mode network; PMN, parietal memory network; ROI, region‐of‐interest

As displayed in Figure 2 and Table 2, voxel‐wise analysis of variance revealed significant group difference among the three groups (*p* < .05, FWE corrected) in the core regions of PMN including bilateral precuneus (lPCU/rPCU) and left posterior cingulate cortex (lPCC), and in the core regions of AN including right Heschl's gyrus (rHG), right superior temporal gyrus (rSTG), and right posterior superior temporal gyrus (rpSTG; see Table 2 for details). As shown in Figure 2, the regions exhibiting significant group difference were not inside the core regions of DMN. Post‐hoc analysis revealed that both AH− and AH+ significantly diminished in the overall functional connectivity of the rHG (for AH−, *t*[91] = 2.26, *p* = .026; for AH+, *t*[84] = 4.10, *p* < .001), rSTG (for AH−, *t*[91] = 4.26, *p* < .001; for AH+, *t*[84] = 2.00, *p* = .049), rpSTG (for AH−, *t*[91] = 3.51, *p* < .001; for AH+, *t*[84] = 3.01, *p* = .0035), lPCC (for AH−, *t*[91] = 3.38, *p* = .0011; for AH+, *t*[84] = 3.76, *p* < .001), and lPCU (for AH−, *t*[91] = 2.84, *p* = .0056; for AH+, *t*[84] = 3.98, *p* < .001). Only AH+ displayed attenuated overall functional connectivity in rPCU compared to both HC (*t*[84] = 5.30, *p* < .001) and AH− (*t*[57] = 3.36, *p* = .0014).

**Table 2:** Group difference in voxel‐wise analysis of variance within PMN and AN

Location	Hemisphere	BA	Voxel size	MNI coordinates			Peak *F*/*p* value^a^
				*x*	*y*	*z*	
*AN*							
Heschl's gyrus	R	41	21	51	−21	12	8.39/.032
Superior temporal gyrus	R	22	14	69	−18	0	8.54/.029
Posterior superior temporal gyrus	R	42	8	60	−30	9	9.15/.030
*PMN*							
Posterior cingulate gyrus	L	23	62	0	−33	24	9.73/.009
Precuneus	R	7	36	21	−60	33	9.57/.020
Precuneus	L	7	6	−6	−78	51	8.15/.038

Abbreviations: AN, auditory network; BA, Brodmann area; PMN, parietal memory network.

a The *p*‐values are FWE corrected.

### 3.4 ROI‐wise functional connectivity analyses

**Figure 3:**
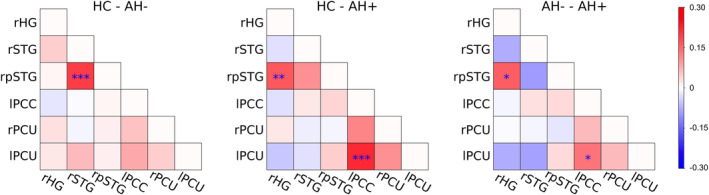
The ROI‐wise functional connectivity difference between HC/AH−, HC/AH+, and AH−/AH+. The color represents the mean functional connectivity difference between the two groups and the asterisks indicate the statistical difference (*<.05, **<.01, ***<.001). AH+ shows significant difference from both HC and AH− in the functional connectivity of rHG and rpSTG in AN as well as that of lPCC and lPCU in PMN. AH, auditory hallucination; AN, auditory network; PMN, parietal memory network; rHG, right Heschl's gyrus; ROI, region‐of‐interest; rpSTG, right posterior superior temporal gyrus

We further examined correlations among the time courses of the above ROIs in PMN and AN, and identified different patterns of functional connectivity across the three groups (Figure 3). The AH+ group exhibited significant decrease in functional connectivity between rHG and rpSTG compared to AH− (*t*[57] = 2.59, *p* = .012) and HC (*t*[84] = 3.12, *p* = .0024) and between lPCC and lPCU (to AH−: *t*(57) = 2.31, *p* = .024; to HC: *t*(84) = 3.81, *p* < .001). Besides, compared to HC, AH− displayed significantly lower functional connectivity between rSTG and rpSTG (*t*[91] = 3.55, *p* < .001) while AH+ did not show statistical difference from HC (*t*[84] = 1.81, *p* = .073). The functional connectivity between the ROIs from AN and PMN did not show significant intergroup difference.

### 3.5 Clinical and cognitive correlations in AH+

**Figure 4:**
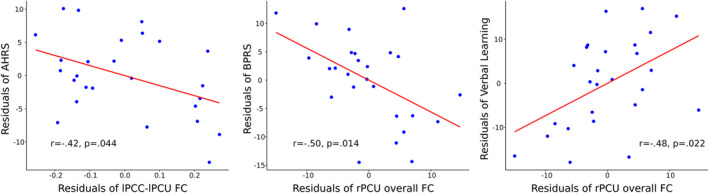
Scatter plots depicting partial correlations between functional connectivity metrics and clinical/cognitive measures in the AH+ group after controlling age, gender, and education years. For the purpose of visualization, the horizontal axes represent residuals of the connectivity metrics after regressing out the above covariates, and the vertical axes represent residuals of the clinical/cognitive measures after regressing out the above covariates. FC, functional connectivity

The above analyses revealed four functional connectivity metrics that exhibited abnormality specific to the AH+ group, including the overall functional connectivity of PMN, the overall functional connectivity of rPCU, the rHG‐rpSTG functional connectivity, and the lPCC–lPCU functional connectivity. As an exploratory analysis, correlations between these metrics and clinical and cognitive measures were computed with age, gender, and education years as covariates in the AH+ group. As shown in Figure 4, the lPCC–lPCU functional connectivity was negatively correlated with AHRS scores (*r*[21] = −.42, *p* = .044), and the overall functional connectivity of rPCU was significantly correlated with the total score of BPRS (*r*[21] = −.50, *p* = .014). For correlations with the memory domains of MCCB (working memory, verbal learning, and visual learning), the overall functional connectivity of rPCU (*r*[21] = 0.48, *p* = .022) was positively correlated with verbal learning domain.

## 4 DISCUSSION

With a sample of first‐episode drug‐naïve schizophrenia or schizophreniform disorder, we found abnormalities in PMN are relevant to AHs. In the network‐wise analysis, we found that the overall functional connectivity of PMN decreased only in AH+; in the voxel‐wise analysis, we further localized the deficits in PMN; our ROI‐wise analysis additionally showed the functional connectivity between lPCC and lPCU decreased only in AH+. We also showed that functional connectivity abnormalities in AH+ further displayed significant correlations with clinical or cognitive measures. In contrast, we failed to find abnormal functional connectivity of DMN in either AH− or AH+ patients. These findings suggest that the separation between PMN and DMN is important in the search for neuroimaging relevance of AHs.

In previous studies, reduced functional connectivity among auditory regions was consistently observed in patients with schizophrenia (Chen et al., 2015; Kaufmann, Skåtun, Alnæs, Doan, & Westlye, 2015). This study showed disintegration in the AN as a general deficit in schizophrenia regardless of the existence of AH symptoms. Furthermore, we showed reduced rHG–rpSTG connectivity in AH+ but not AH− patients, suggesting the dysconnectivity within right STG may contribute to AHs. These findings replicate previous reports and help to validate our methodology.

Based on the delineation of DMN and PMN using ICA, the present work found that the dysfunction associated with AHs was restricted to PMN but not DMN. Current result may attribute to the heterogeneity within the posteromedial cortex of human (the posterior part of DMN), which has been implicated by both structural (Buckner et al., 2008; Leech & Sharp, 2014; Margulies et al., 2009) and functional (Andrews‐Hanna, Reidler, Sepulcre, Poulin, & Buckner, 2010; Cauda et al., 2010; Dastjerdi et al., 2011; Zhang & Li, 2012) studies regarding the DMN. Although the precuneus and PCC have been frequently mentioned as parts of DMN, functional heterogeneity of above regions (Cha, Jo, Gibson, & Lee, 2017; Luo et al., 2019) indicates the possibility that these regions can involve in other functional networks, such as precuneus network (Shirer, Ryali, Rykhlevskaia, Menon, & Greicius, 2012) or control network (Kim, 2018; Yeo et al., 2011) by different functional parcellations. Therefore, the regions “precuneus” and “PCC” are not naturally equivalent to parts of DMN.

In this study, we defined DMN according to the canonical definition that includes medial prefrontal cortex, precuneus, and posterior cingulate cortex and bilateral inferior parietal lobules, which are always co‐existent according to previous studies (Raichle, 2015). The PMN exhibited a distinct intrinsic functional connectivity pattern from the canonical DMN despite its spatial adjacency to DMN, and had relatively low functional connectivity not only to the medial frontal cortex, but also the posterior part of the DMN (Figure S1). Our previous studies also revealed that this network exhibited a different interindividual variability pattern from the canonical DMN in lifespan development (Yang et al., 2014), Alzheimer's disease and movie watching (Deng et al., 2019; Hu et al., 2019). If this network differs in many aspects from the canonical DMN, it seems more beneficial to refer to this network with an informative name. By separating the PMN from the DMN in this study, we further refined the cognitive hypothesis of aberrant attention in coordinating internal and external agents that may contribute to AHs. Up to date, a few studies have implied the relevance of the precuneus and PCC to AHs (Allen et al., 2012; Nenadic, Smesny, Schlosser, Sauer, & Gaser, 2010; Wolf et al., 2011), but the hypothesis of functional separation between the PMN and the DMN in contributing to AH symptoms is proposed for the first time.

In a clinical aspect, deficits in pivotal regions of PMN were associated with severity of AHs as well as memory domains of MCCB (verbal learning) among AH+ patients. Though the correlations were not corrected for multiple comparisons and should be viewed as exploratory analyses, the current results emphasize the effortful involvement of PMN in forming verbal‐related memory and echoes dysfunction of verbal memory in AH (Allen et al., 2012). In accordance with our study, a task‐fMRI study on noise‐masking speech recognition reported declined functional connectivity of the STG with the bilateral precuneus in schizophrenia, suggesting increased vulnerability to process masked verbal information under cocktail‐party‐listening conditions (Li et al., 2017). Mashal's group revealed increased connection between right precuneus and right posterior STG during novel metaphor comprehension in patients with schizophrenia, proposing the over‐integration of language and non‐language brain regions during more effortful processes of verbal task in patients (Mashal, Vishne, & Laor, 2014). Their results partly support our hypothesis that alterations in PMN are involved in the dysfunction of auditory or language processing in schizophrenia. The reason that our data did not discover aberrant interaction among core regions of AN and PMN in resting‐state may be due to the absence of verbal‐related task design in our study.

Since persistent AHs could lead to ongoing disability and distress for schizophrenia, our findings, if validated, may provide an effective target for clinical interventions of AHs, especially for the drug‐resistant AHs. Previous evidence‐based data showed that the efficacy of inhibitory rTMS over temporoparietal junction for treatment of AH only demonstrated moderate effect (Lefaucheur et al., 2014). According to the findings in this study, we speculate that excitatory rTMS may be applied to the core regions of PMN to relieve the disturbing symptom. In addition, the alterations in PMN in longitudinal studies should be investigated to examine its association with the clinical outcomes of patients with AH, and in turn to help optimize the treatment strategy for AH.

A limitation of the study is that the AH states of the participants during scanning were not recorded or controlled, so that we were not able to identify the state‐related biomarkers. With the nature of a trait design, this study cannot examine the state‐related abnormalities either in PMN or DMN, and therefore our results did not exclude the possibility that the absence of between‐group difference for the DMN may be due to specific state‐features of DMN regarding AH. A paradigm that better constrains mental states and a post‐hoc questionnaire should be used in the future studies to overcome this issue. Alternatively, the connectivity in auditory or language processing could be further evaluated in AH+ patients with task‐fMRI study.

## 5 CONCLUSION

In conclusion, this study proposed and examined a hypothesis that deficits in the intrinsic activity of PMN contributed to the AH symptoms. The correlation between the deficits in PMN and verbal memory cognitive performance helps to interpret the involvement of PMN in AH symptoms and links brain imaging findings to cognitive theories of AHs. Further, the findings demonstrate the separation between PMN and DMN is important in the search for neuroimaging relevance of AHs. Future works should focus on delineating the detailed deficit in the PMN and confirming treatment effects via intervention on PMN activity.

## CONFLICT OF INTEREST

The authors report no biomedical financial interests or potential conflicts of interest.

## Supporting information


**Appendix S1**: Supplementary Materials

## Data Availability

I confirm that my article contains a Data Availability Statement even if no data is available (list of sample statements) unless my article type does not require one. I confirm that I have included a citation for available data in my references section, unless my article type is exempt.
