# Supplementary material for: Parietal memory network and default mode network in first‐episode drug‐naïve schizophrenia: Associations with auditory hallucination
Source: Hum Brain Mapp. 2020 Feb 29;41(8):1973–84. doi: 10.1002/hbm.24923 (PMC7267906; doi:10.1002/hbm.24923)
Supplement: Supplementary file 1 — Appendix S1: Supplementary Materials [file HBM-41-1973-s001.docx]

Supplementary Materials


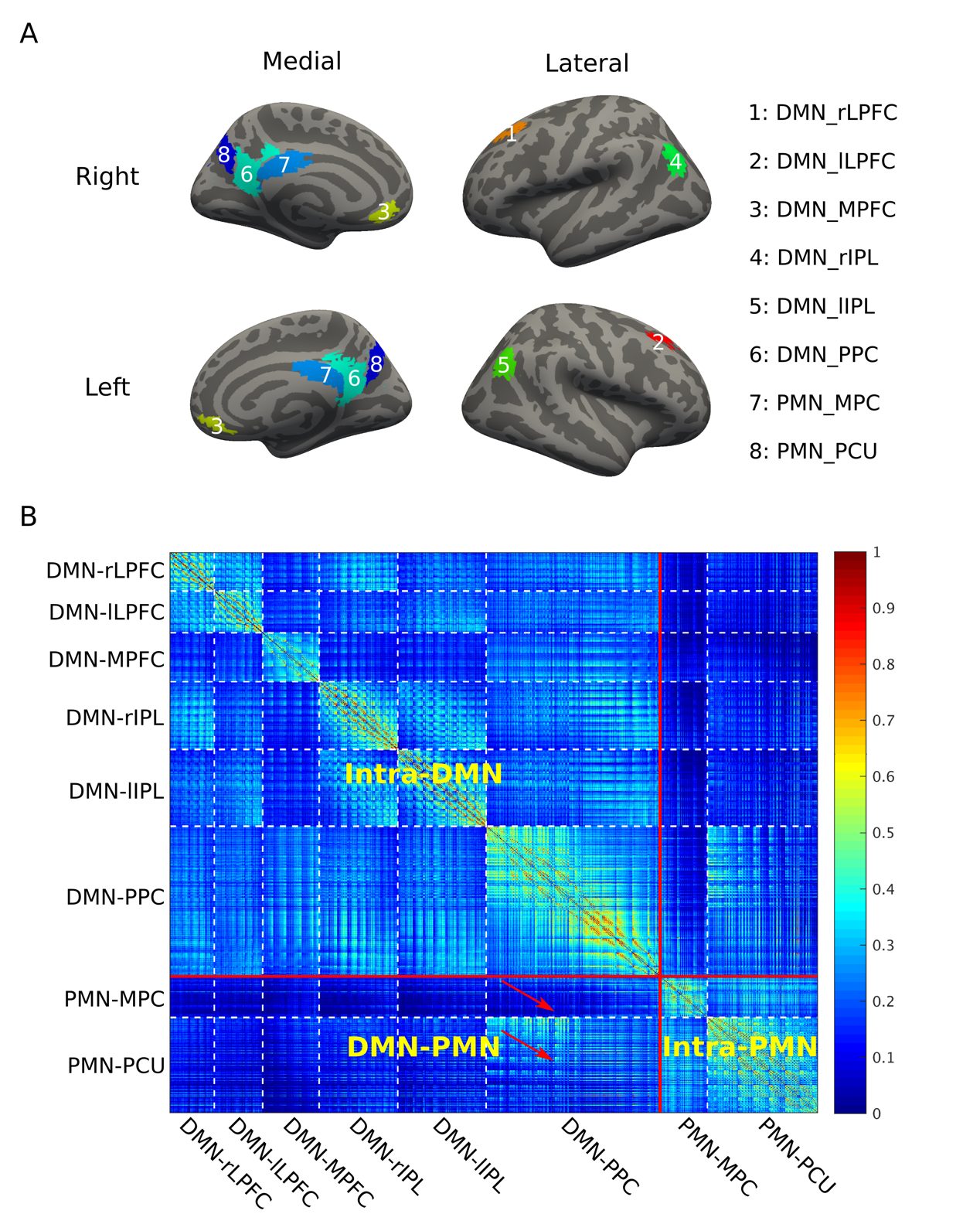


Figure 1. The average voxel-wise functional connectivity of PMN and DMN in the HC group. (A) The ROI masks were created for PMN and DMN: The PMN and DMN group-level component maps were converted to Z-scores and thresholded at Z=5 and Z=3 respectively. The thresholds were chosen to resemble the patterns of PMN and canonical DMN commonly reported in literature and have minimal overlaps. The overlaps (43 voxels) were further removed from each network. The DMN included six ROIs: bilateral lateral prefrontal cortex (rLPFC/lLPFC), medial prefrontal cortex (MPFC), bilateral inferior parietal lobule (rIPL/lIPL) and precuneus/posterior cingulate cortex (PPC), while the PMN included two ROIs: middle/ posterior cingulate cortex (MPC) and precuneus (PCU). The ROI masks were projected onto the fsaverage surface for visualization and shown in different colors. (B) The average connectivity pattern of PMN and DMN in HC group: the voxel-wise Pearson correlation coefficients (Fisher’s Z transformed) were calculated within these ROIs for each subject and averaged in the HC group. It should be noted (indicated using red arrows) that the PMN_MPC and DMN_PPC were spatially adjacent but have weak connections, while the PMN_PCU and DMN_PPC has some strong connections, indicating potential functional overlaps. Furthermore, we compared the intra-network and inter-network averaged functional connectivity using a paired t-test in the HC group. The mean (standard deviation) functional connectivity were 0.31 (0.085) and 0.23 (0.069) for the intra-PMN and intra-DMN, and 0.11 (0.046) for DMN-PMN respectively. The intra-network and inter-network difference were significantly different (p < .0001).


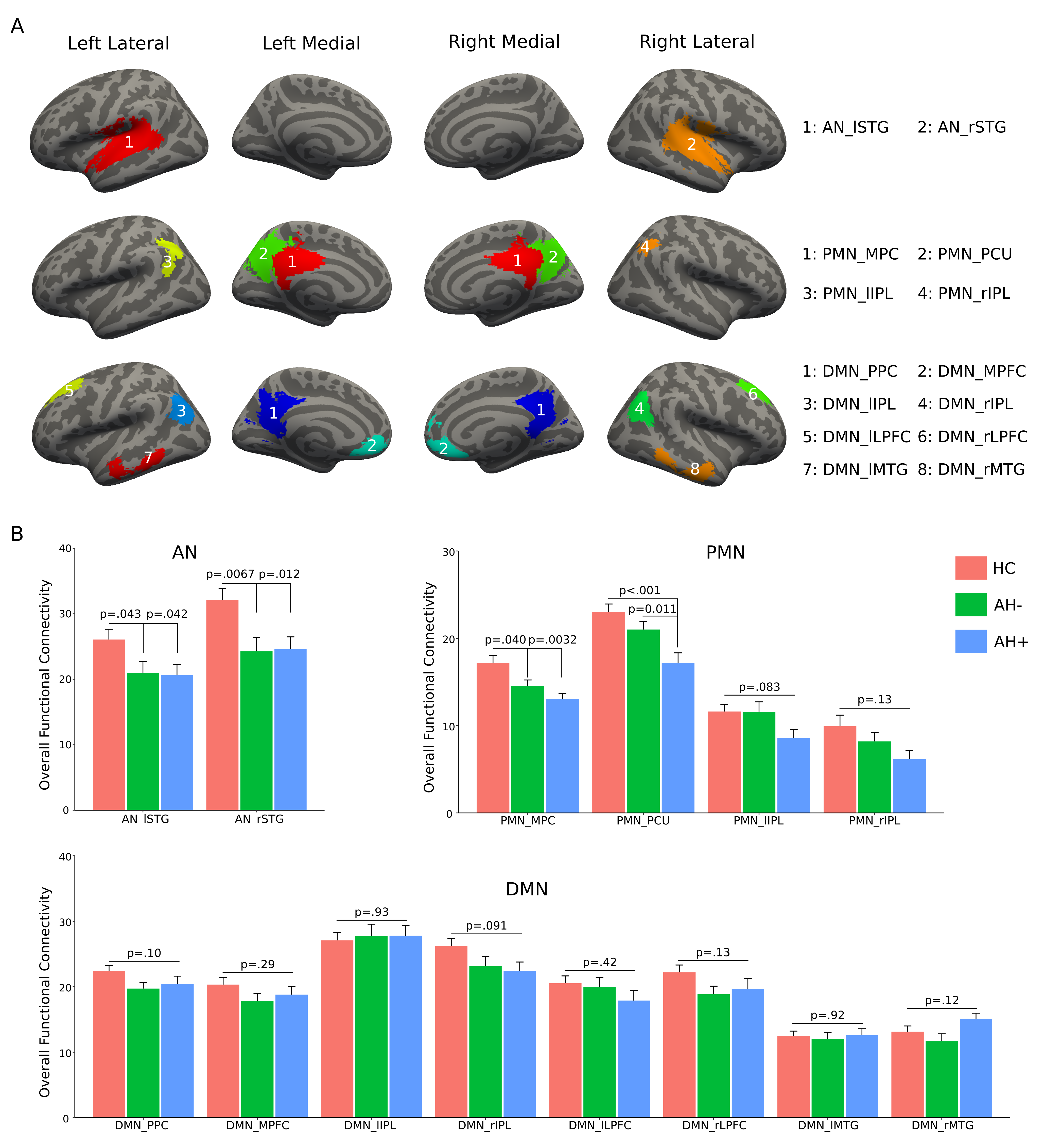


Figure 2. The analysis of major nodes for AN, PMN and DMN. (A) The nodes were delineated for AN, PMN and DMN: the group-level component maps were thresholded at a local false discovery rate of p<0.05 using Gaussian mixture model and the clusters with a minimum of 200 voxels were treated as nodes for each network. For PMN, the large medial cluster was further split into two parts based on the precunues/posterior cingulate boundary. The AN included two nodes: bilateral superior temporal gyrus (rSTG/lSTG), the PMN included four nodes: middle/posterior cingulate cortex (MPC), precuneus (PCU), and bilateral inferior parietal lobules (rIPL/lIPL), and the DMN included eight ROIs: precuneus/posterior cingulate cortex (PPC), medial prefrontal cortex (MPFC), bilateral inferior parietal lobule (rIPL/lIPL), bilateral lateral prefrontal cortex (rLPFC/lLPFC) and bilateral middle temporal gyrus (rMTG/rMTG). The nodes were projected onto the fsaverage surface for visualization and shown in different colors. (B) The group comparison results for major nodes. For AN, the HC showed significant higher overall functional connectivity than both AH- and AH+ groups in all nodes; for PMN, both HC and AH- groups showed significantly higher overall functional connectivity than AH+ in the PCU node, and the HC group showed significantly higher overall functional connectivity than both AH- and AH+ groups in the MPC node, while there were no significant difference in the bilateral IPL nodes among three groups; for DMN, there were no significant difference in all eight nodes.

Table S1 Comparisons in All Domains of MCCB Across Three Groups

| MCCB domains | Schizophrenic subgroups | | Healthy controls  (SD) (N = 59) | Statistical test (p value)^b^ |
| --- | --- | --- | --- | --- |
|  | AH- patients (SD) (N = 31) | AH+ patients (SD) (N = 26) |  |  |
| Speed of processing ^a^ | 37.71(12.84) | 41.38(12.55) | 49.22(10.62) | F = 10.97(p < 0.001) |
| Attention/vigilance ^a^ | 38.74(11.43) | 40.88(13.10) | 47.85(8.89) | F = 8.75(p < 0.001) |
| Working memory ^a^ | 34.65(13.22) | 38.85(11.59) | 44.14(11.51) | F = 6.64(p = 0.002) |
| Verbal learning ^a^ | 37.10(13.37) | 37.04(11.47) | 42.64(9.57) | F = 3.65(p = 0.029) |
| Visual learning ^a^ | 41.23(14.00) | 45.35(14.65) | 49.22(10.12) | F = 4.35(p = 0.015) |
| Reasoning and problem solving ^a^ | 40.19(14.90) | 43.19(10.18) | 49.46(10.04) | F = 7.24(p = 0.001) |
| Social cognition ^a^ | 38.81(12.64) | 44.58(13.83) | 39.97(9.50) | F = 2.04(p = 0.135) |
| Overall composite score ^a^ | 34.74(13.59) | 37.62(13.16) | 45.08(9.82) | F = 9.06(p < 0.001) |

Abbreviations: AH-, schizophrenic or schizophreniform patients without auditory hallucinations; AH+, schizophrenic or schizophreniform patients with auditory hallucinations; MCCB, MATRICS Consensus Cognitive Battery.

^a^ Data are presented as mean(SD)，^b^ p values are in parentheses

The data of 3 subjects were not available (one for HC group and two for AH- group)

Table S2. The statistical analysis for the major nodes of AN, PMN and DMN.

| Node | HC | AH- | AH+ | F-test (p value)^b^ | HC vs. AH- (p value)^b^ | HC vs. AH+(p value)^b^ | AH- vs. AH+(p value)^b^ |
| --- | --- | --- | --- | --- | --- | --- | --- |
| AN_lSTG ^a^ | 26.07 (12.21) | 20.98 (9.83) | 20.64 (8.20) | F(2,116)=3.50 (0.034) | t(91)=2.06 (0.043) | t(84)=2.07(0.042) | t(57)=0.14(0.89) |
| AN_rSTG ^a^ | 32.14 (13.54) | 24.28 (12.22) | 24.57 (9.74) | F(2,116)=5.75(0.0042) | t(91)=2.77(0.0067) | t(84)=2.57 (0.012) | t(57)=-0.10(0.92) |
| PMN_MPC ^a^ | 17.18 (6.63) | 14.58 (3.75) | 13.04 (3.12) | F(2,116)=6.26 (0.0026) | t(91)=2.08 (0.040) | t(84)=3.03(0.0032) | t(57)=1.68(0.099) |
| PMN_PCU ^a^ | 23.02 (7.02) | 21.01 (5.33) | 17.18 (5.90) | F(2,116)=7.67(<0.001) | t(91)=1.43 (0.16) | t(84)=3.71(<0.001) | t(57)=2.62(0.011) |
| PMN_lIPL ^a^ | 11.62 (6.28) | 11.59 (6.50) | 8.57 (4.91) | F(2,116)=2.54(0.083) | - | - | - |
| PMN_rIPL ^a^ | 9.94 (9.75) | 8.19 (5.93) | 6.16 (4.90) | F(2,116)=2.12(0.13) | - | - | - |
| DMN_PPC ^a^ | 22.39 (6.47) | 19.72 (5.43) | 20.43 (6.08) | F(2,116)=2.31(0.10) | - | - | - |
| DMN_MPFC ^a^ | 20.33 (8.48) | 17.82 (6.42) | 18.79 (6.48) | F(2,116)=1.25 (0.29) | - | - | - |
| DMN_lIPL ^a^ | 27.08 (9.24) | 27.70 (10.65) | 27.80 (8.01) | F(2,116)=0.075 (0.93) | - | - | - |
| DMN_rIPL ^a^ | 26.21 (8.97) | 23.14 (8.55) | 22.43 (6.81) | F(2,116)=2.45 (0.091) | - | - | - |
| DMN_lLPFC ^a^ | 20.53 (8.70) | 19.91 (8.52) | 17.89 (7.95) | F(2,116)=0.88 (0.42) | - | - | - |
| DMN_rLPFC ^a^ | 22.20 (8.67) | 18.86 (7.04) | 19.62 (8.48) | F(2,116)=2.06(0.13) | - | - | - |
| DMN_lMTG ^a^ | 12.46 (5.84) | 12.04 (5.75) | 12.60 (4.98) | F(2,116)=0.086(0.92) | - | - | - |
| DMN_rMTG ^a^ | 13.13 (6.76) | 11.68 (6.46) | 15.10 (4.39) | F(2,116)=2.19 (0.12) | - | - | - |

Abbreviations: AH-, schizophrenic or schizophreniform patients without auditory hallucinations; AH+, schizophrenic or schizophreniform patients with auditory hallucinations;.

^a^ Data are presented as mean(SD)，^b^ p values are in parentheses
